# DNA repair in cancer development and aging

**DOI:** 10.18632/aging.203656

**Published:** 2021-10-26

**Authors:** Hua Zhao, Bernard F. Fuemmeler, Jie Shen

**Affiliations:** 1 Department of Family Medicine and Population Health, School of Medicine, Virginia Commonwealth University, Richmond, VA 23284, USA; 2 Department of Health Behavior and Policy, School of Medicine, Virginia Commonwealth University, Richmond, VA 23284, USA

**Keywords:** cancer, aging, DNA repair, phenotypic assays, disease prevention

Genomic instability is one of the most vital aspects that lead to carcinogenesis [1]. DNA repair performs the essential role of repairing DNA damages from exposure to numerous endogenous and exogenous carcinogens, thus maintaining genomic stability. Therefore, suboptimal DNA repair capacity is deemed as a critical driving force behind cancer development. In addition, DNA repair also plays a crucial role in aging [2]. In tissues, the accumulation of DNA damage caused by suboptimal DNA repair may lead to cellular senescence and function decline and promote biological aging.

Given the significance of DNA repair in both cancer development and aging, it has been hypothesized that individuals with suboptimal DNA repair capacity may predispose to increased risks of cancer and biological aging. The most robust evidence to support the hypothesis is from the observations in a variety of rare inherited human syndromes (e.g., ataxia telangiectasia, Werner syndrome, Bloom Syndrome, Fanconi anemia, xeroderma pigmentosum, etc.) caused by rare germline mutations in DNA repair genes, which exhibit a premature aging phenotype and have an increased cancer risk [3]. From a public health perspective, the ability to identify individuals who have suboptimal DNA repair capacity is important because those individuals may have an increased risk of cancers and biological aging, and thereby, they will be potentially benefited from cancer and aging preventive strategies.

However, whether such assumption can be established in the general population remains to be determined. Both genotypic and phenotypic approaches have been proposed to assess DNA repair capacity. Genetic association studies have identified several common and rare genetic variations in DNA repair genes that may modify DNA repair capacity [4]. However, those variants in DNA repair genes may only account for a small proportion of DNA repair variability in the general population. Furthermore, the identified common variants usually have minimal effect size, and their functional relevance to DNA repair capacity is lagging. Although the explosive usage of next-generation sequencing has resulted in the continuous discovery of rare novel variants, the pathogenicity of these variants and the risk they convey are still most challenging to determine [5]. Thus, to date, the application of genetic assays to assess DNA repair capacity in the general population is still limited. Large-scale DNA sequencing analysis to discover additional genetic variants and the corresponding functional analysis to ascertain their functional relevance to DNA repair capacity is needed.

In contrast to genotypic assays, the phenotypic assays, from gene expression, molecular function to cellular activity, can be instrumental in assessing DNA repair capacity because they are sensitive, robust, and, more importantly, biologically relevant. One advantage of phenotypic markers is that they can measure effects from a combination of genes and their functional variants, regardless of whether they are known or novel. The classic examples include various types of DNA damage and repair assays [6], such as mutagen sensitivity, micronucleus frequencies, chromosomal radio-sensitivity, and chromosomal damage assays, all of which have been used in molecular cancer epidemiologic studies. Recently, we developed a phenotypic assay to measure homologous recombination repair capacity in peripheral blood lymphocytes [7]. In our breast cancer study, we found that DNA repair capacity was significantly lower in cases than in controls (P<0.001), and decreased HRR capacity was associated with an increased risk of breast cancer. However, several limitations have hindered the application of those functional assays on a large scale. For example, many of the assays require large amounts of freshly collected pure cell types. In addition, they only address the repair capacity of a subset of specific lesions. Also, the reliability and validity of its results may be affected by experimental conditions [8]. Thus, more efforts are needed to develop new phenotypic assays or enhance existing ones to address those concerns.

In the era of precision prevention, tools are needed to help us to identify the right individuals and match them with the right preventive strategies. Given the significant relevance of DNA repair to genome integrity and thereby cancer development and aging, the idea of assessing DNA repair in precision prevention is particularly appealing. Therefore, it would be of continued interest to further develop both genotypic and phenotypic DNA repair assays an incorporate them in the precision prevention framework.

## References

[1] Negrini S, et al. Nat Rev Mol Cell Biol. 2010; 11:220–28. 10.1038/nrm2858 20177397

[2] Chen Y, et al. Ageing Res Rev. 2020; 64:101154. 10.1016/j.arr.2020.101154 32977059

[3] Knoch J, et al. Eur J Dermatol. 2012; 22:443–55. 10.1684/ejd.2012.1654 22436139

[4] Abdel-Rahman SZ, El-Zein RA. Biomarkers. 2011; 16:393–404. 10.3109/1354750X.2011.577237 21595606 PMC3142279

[5] Rapposelli IG, et al. BMC Cancer. 2021; 21:611. 10.1186/s12885-021-08368-5 34034685 PMC8152298

[6] Berwick M, Vineis P J Natl Cancer Inst. 2000; 92:1537. 10.1093/jnci/92.18.1537 10995816

[7] Shen Jie et al. Carcinogenesis. 2020; 41:1363–67. 10.1093/carcin/bgaa081 32692853 PMC7566366

[8] Collins AR Biochim Biophys Acta. 2014; 1840:794–800. 10.1016/j.bbagen.2013.04.022 23618695

